# Balanced Intense Exercise Training Induces Atrial Oxidative Stress Counterbalanced by the Antioxidant System and Atrial Hypertrophy That Is Not Associated with Pathological Remodeling or Arrhythmogenicity

**DOI:** 10.3390/antiox10030452

**Published:** 2021-03-15

**Authors:** Attila Oláh, Bálint András Barta, Alex Ali Sayour, Mihály Ruppert, Eszter Virág-Tulassay, Julianna Novák, Zoltán V. Varga, Péter Ferdinandy, Béla Merkely, Tamás Radovits

**Affiliations:** 1 Heart and Vascular Center, Semmelweis University; Városmajor str. 68, 1122 Budapest, Hungary; barta.balint_andras@med.semmelweis-univ.hu (B.A.B.); sayour.alex_ali@med.semmelweis-univ.hu (A.A.S.); ruppert.mihaly@med.semmelweis-univ.hu (M.R.); tulassay.eszter@gmail.com (E.V.-T.); merkely.bela@med.semmelweis-univ.hu (B.M.); radovits.tamas@med.semmelweis-univ.hu (T.R.); 2 HCEMM-SU Cardiometabolic Immunology Research Group, Semmelweis University; Nagyvárad tér 4, 1089 Budapest, Hungary; junoposta@gmail.com (J.N.); varga.zoltan@med.semmelweis-univ.hu (Z.V.V.); 3 Department of Pharmacology and Pharmacotherapy, Semmelweis University; Nagyvárad tér 4, 1089 Budapest, Hungary; peter.ferdinandy@pharmahungary.com; 4 Pharmahungary Group, 6722 Szeged, Hungary

**Keywords:** athlete’s heart, myocardial antioxidant system, in vivo electrophysiology, atrial remodeling

## Abstract

Although regular exercise training is associated with cardiovascular benefits, the increased risk of atrial arrhythmias has been observed after vigorous exercise and has been related to oxidative stress. We aimed at investigating exercise-induced atrial remodeling in a rat model of an athlete’s heart and determining sex-specific differences. Age-matched young adult rats were divided into female exercised, female control, male exercised, and male control groups. After exercised animals completed a 12-week-long swim training protocol, echocardiography and in vivo cardiac electrophysiologic investigation were performed. Additionally, atrial histological and gene expression analyses were carried out. Post-mortem atrial weight data and histological examination confirmed marked atrial hypertrophy. We found increased atrial gene expression of antioxidant enzymes along with increased nitro-oxidative stress. No gene expression alteration was found regarding markers of pathological remodeling, apoptotic, proinflammatoric, and profibrotic processes. Exercise training was associated with a prolonged right atrial effective refractory period. We could not induce arrhythmias by programmed stimulation in any groups. We found decreased expression of potassium channels. Female gender was associated with lower profibrotic expression and collagen density. Long-term, balanced exercise training-induced atrial hypertrophy is not associated with harmful electrical remodeling, and no inflammatory or profibrotic response was observed in the atrium of exercised rats.

## 1. Introduction

Each physical exercise bout involves a volume or pressure overload that superimposes the cardiac chambers. Regular, intense exercise training induces a complex adaptation of the cardiovascular system, and plentiful recent studies demonstrated dose-dependency of exercise-induced alterations in the heart [1,2,3]. Physical activity decreases the risk of cardiovascular disease by reducing the burden of co-morbidities and prevents the aging of the myocardial tissue [4]. Moreover, advanced levels of exercise training, which lead to physiological ventricular hypertrophy, are associated with improved cardiovascular performance [5]. Atrial dilation is a common feature in athletes and can be characterized as a benign adaptation with preserved atrial reservoir function, contrary to pathological conditions [6].

While the cardiovascular benefits of regular, moderate exercise are beyond question, long-standing vigorous exercise may be associated with transient functional deterioration, as well as with sustained adverse electrical and structural remodeling [1,7,8]. Among other pathological consequences, considerable evidence supports an increased incidence of atrial fibrillation (AF) in athletes undergoing long-term, high-intensity training [9,10]. Indeed, the thin wall of the atria might be vulnerable to sustained volume overload associated with prolonged endurance exercise. The potential main factors leading to pathological atrial alterations are a local inflammatory response to excessive oxidative stress, increased mechanical stress-induced myocardial remodeling due to volume overload, excessive vagal tone enhancement, and direct electrical modification of cardiomyocytes [10,11]. Indeed, transient oxidative stress and the compensatory overexpression of the antioxidant system play a central role in exercise-induced myocardial alterations.

Cardiovascular response to intense exercise training might fundamentally differ between male and female athletes [5,12]. The prevalence of atrial fibrillation is usually higher in men compared to age-matched women. Moreover, recent literature data about the relationship between atrial fibrillation and exercise suggested that there may be a sex-specific effect on AF occurrence in highly trained individuals [9,13]. While the prevalence of AF shows a negative correlation with the dose of sports activity in women, this exercise-dosage curve is associated with a J shape pattern in men, meaning that intense training is associated with increased AF risk only in male individuals [9]. This sex-related dichotomy suggests different regulatory mechanisms in the atria of males and females.

However, there are many gaps in evidence related to the mechanisms of AF in endurance athletes. Therefore we aimed to investigate exercise training-associated structural and electrical atrial alterations in a rat model, where a balanced training program resulted in relevant ventricular hypertrophy. We also focused on oxidative stress and antioxidant enzyme expression, as well as investigated the sex-specific response. Therefore, both male and female rats were involved in this experimental investigation.

## 2. Materials and Methods

### 2.1. Animals

This study was carried out in accordance with the principles of the Guide for the Care and Use of Laboratory Animals provided by the National Institute of Health (NIH Publication No. 86–23, revised 1996.) and the European Union (EU) Directive 2010/63/EU. The protocol was approved by the Ethical Committee for Animal Experimentation, Semmelweis University, Budapest (PEI/001/2374-4/2015). All animals received high-quality care.

Young adult, age-matched, 57-61 days old male (*n* = 36) and female (*n* = 36) Wistar rats were housed in standard rat cages at a constant room temperature (22 ± 2 °C) and humidity with a 12:12-h light–dark cycle. The animals were allowed access to standard laboratory chow and water ad libitum during the whole experimental period.

### 2.2. Experimental Groups and Setting

After acclimation, male and female rats were randomly assigned to matched control (Co) or exercised (Ex) groups:

- Male control (MCo, *n* = 18)
- Male exercised (MEx, *n* = 18)
- Female control (FCo, *n* = 18)
- Female exercised (FEx, *n* = 18).

After completion of the 12-week long exercise training period, rats underwent echocardiography. In vivo electrophysiological studies were carried out in 12–12 animals, followed by cardiac excision and atrial formalin preservation for histology. The atrial samples of 6–6 rats from each group were snap-frozen in liquid nitrogen to be used for gene expression analysis.

### 2.3. Exercise Training Protocol—Rat Model of Physiological Cardiac Hypertrophy

For long-term exercise training, both male and female rats swam for a total period of 12 weeks, for 200 min/day, 5 days a week, as previously described [14]. The protocol included an adaptation phase, where the duration of swimming was increased 15 min every second training day from a basic 15 min on the first day until achieving the maximal 200 min/day. The water temperature was maintained at 30–32 °C during the exercise session. Untrained control rats were placed into the water for 5 min each day during the 12-week training program. In vivo measurements were performed at least 6 h, but not more than 12 h after the last exercise session.

### 2.4. Echocardiography

At the completion of the swimming training program, left ventricular (LV) morphological alterations were observed by echocardiography using a 13 MHz linear transducer (12L-RS, GE Healthcare, Horten, Norway) connected to a commercially available system (Vivid i, GE Healthcare), as described earlier [15]. Rats were anesthetized using isoflurane (induction dose: 5%, maintenance dose: 2%). Animals were placed on controlled heating pads, and the core temperature was maintained at 37 °C. Standard two-dimensional and M-mode long- and short-axis (at mid-papillary level) images were acquired. On two-dimensional recordings of the short-axis at the mid-papillary level, LV anterior (AWT) and posterior (PWT) wall thickness in diastole (index: d) and systole (index: s), as well as LV end-diastolic (LVEDD) and end-systolic diameter (LVESD), were measured. LV mass was determined according to the following formula: LVmass = [(LVEDD + AWTd + PWTd)^3^-LVEDD^3^] × 1.04. To calculate LV mass index, we normalized the LV mass values to the tibial length (TL) of the animal.

### 2.5. In Vivo Right Atrial Electrophysiology

The procedure was performed under general anesthesia with 2%-isoflurane. Body temperature was strictly maintained between 36.7 °C and 37.3 °C. An incision was made in the right supraclavicular region, and a 1.6 F octapolar electrode catheter (Millar EPR-802; Millar Instruments, Houston, TX, US) was placed in the right internal jugular vein. The catheter was advanced to the right atrium using electrogram guidance and pacing capture to verify intracardiac position. A computer-based data acquisition system (PowerLab 16/30; ADInstruments, Colorado Spring, CO, USA) was used to record a 1-lead body surface electrocardiogram (ECG) (lead II) and up to 4 intracardiac bipolar electrograms (LabChart Pro software v7; AD Instruments). Bipolar pacing through the distal electrodes was carried out with an impulse generator (STG3008-FA, Multi Channel Systems, Reutlingen, Germany) triggered by special software (MC Stimulus II, Multi Channel Systems). The pacing capture intensity threshold was explored, and the double value of threshold intensity was used during pacing protocols.

Sinus node recovery time (SNRT) was measured after applying a 30-s atrial pacing train at a cycle length (CL) of 150 ms. The time interval between the last pacing and the first physiological P-wave is determined as SNRT.

To assess the Wenckebach cycle length (WCL), consecutive steps of 50-beat train stimuli were administered. CL was reduced by 2 ms at each step until an atrioventricular (AV) block was observed. The longest CL inducing Mobitz type-1 AV-block constituted the WCL.

To determine the right atrial refractory period (RAERP), the measurements were continued after 10 min stationary period, paying special attention to the body temperature. RAERP was tested through programmed right-atrial stimulation with a 10-beat train (S1, CL 150 ms) followed by an extra stimulus (S2) that was decreased 1 ms per step until refractoriness. The atrial effective refractory period was defined as the longest coupling interval failing to produce a propagated atrial response.

Atrial arrhythmia inducibility was tested with double extra stimulation (DES) and burst pacing. DES was performed following a 10-beat atrial-pacing train at a CL of 150 ms, followed by one extra stimulus (S2) 10 ms longer than RAERP, while the third extra stimulus (S3) was decreased by 2 ms until refractoriness was reached. Atrial burst pacing trains at 60 and 40 ms CL were applied for 15 and 30 s. AF was defined by >1 s irregular atrial electrograms with an irregular ventricular response. AF was considered non-sustained (nsAF) when it lasted between 1 and 30 s.

### 2.6. Quantitative Real-Time Polymerase Chain Reaction

Whole left and right atrial tissue samples were homogenized in Buffer RLT (Qiagen, Netherlands) using Bertin Precellys 24 Tissue Homogenizer with Bertin Cryolys cooling system (Bertin Technologies, France) to ensure adequate and constant cooling (~0 °C) of samples throughout the procedure. Then, total RNA was isolated using the RNeasy Fibrous Tissue Kit (Qiagen) as per the manufacturer’s protocol. RNA concentration was measured photometrically at 260 nm, while RNA purity was ensured by obtaining 260/280 nm and 260/230 nm optical density ratio of ∼2.0, respectively.

Reverse transcription of RNA to cDNA was conducted with a QuantiTect Reverse Transcription Kit (Qiagen) using 1 μg RNA of each sample and random primers, as per protocol. Then, quantitative real-time polymerase chain reaction (qRT-PCR) was performed on a StepOnePlus RT PCR System (ThermoFisher Scientific, Waltham, MA, USA) using TaqMan Universal PCR MasterMix and TaqMan Gene Expression Assays (ThermoFisher Scientific) for the following targets: atrial natriuretic factor (ANF, assay ID: Rn00561661_m1); Bcl-2 associated X protein (Bax, assay ID: Rn02532082_g1); Bcl-2 (assay ID: Rn99999125_m1); connexin (Cx) 40 (Gja5, assay ID: Rn00570632_m1); Cx43 (Gja1, assay ID: Rn01433957_m1); potassium channels: Kcna5, assay ID: Rn00564245_s1; Kcnd2, assay ID: Rn00581941_m1; Kcnd3, assay ID: Rn04339183_m1; Kcnj2, assay ID: Rn00568808_s1; Kcnj3, assay ID: Rn00434617_m1; matrix metalloproteinase-2 (MMP2, assay ID: Rn01538170_m1), nicotinamide adenine dinucleotide phosphate (NADPH) oxidase 2 (NOX2, assay ID: Rn00576710_m1), superoxide dismutase 2 (SOD-2, assay ID: Rn00690587_g1), transforming growth factor β1 (TGFβ, assay ID: Rn00572010_m1) and tumor necrosis factor α (TNFα, assay ID: Rn99999017_m1). Every sample was quantified in duplicates or triplicates in a volume of 10 μL in each well containing 1 μL cDNA. Data were normalized to the housekeeping glyceraldehyde 3-phosphate dehydrogenase (GAPDH), then to a positive calibrator (a pool of cDNA from all samples of the MCo group) in each case. Accordingly, gene expression levels were calculated using the comparative method (2^−ΔΔCT^).

### 2.7. Histology

Left and right atrial myocardial tissue samples of 6–6 animals per group were removed for histological processing, and then they were fixed in neutral buffered formalin (4%) and embedded in paraffin.

Slices of the left atria were sectioned (5 μm) and processed conventionally for histological examination. After staining these myocardial tissue sections with hematoxylin and eosin (HE), the tissue structure was examined by light microscopy. The transverse, transnuclear diameter of 20 randomly selected cardiomyocytes per animal in the longitudinal orientation of HE stained sections were measured using ImageJ software (National Institutes of Health, Bethesda, MD, USA) and then averaged per animal.

Apoptosis in cardiomyocytes was determined with the terminal deoxynucleotidyl transferase-mediated dUTP nick-end labeling (TUNEL) technique. TUNEL staining was performed using a DeadEnd™ Colorimetric TUNEL System (Promega, Madison, WI, USA) according to the manufacturer’s instruction. Three visual fields of left and right atrial sections were randomly selected in each animal, and TUNEL-positive cells were counted.

To investigate atrial collagen content, the picrosirius red positive area was calculated using ImageJ image analysis software. Three randomly selected left atrium (LA) and right atrium (RA) fields (magnification 200×) were investigated from each–each animal. The fractional area (picrosirius red positive area to total area ratio) was determined on each image, and the mean value of the images represents each animal.

To assess the extent of nitro-oxidative stress, tyrosine nitration was detected in left and right atrial myocardial sections by immunohistochemistry. Paraffin-embedded sections of the myocardium were deparaffinized and hydrated. After antigen retrieval and inactivation of endogenous peroxidase activity with 3% H_2_O_2_ for 10 min, we performed serum blocking. Then slides were immunostained with a rabbit anti-nitrotyrosine (NT) antibody (#06-284, Merck Millipore, MA, USA) at a 1:200 dilution of primary antibody overnight at 4 °C. Specific labeling was detected by incubation for 1 h at room temperature with the secondary antibody (anti-Rabbit HRP) and amplified with phosphate-buffered saline/PBS. Diaminobenzidine/DAB was used as chromogen. Nitrotyrosine positive area was calculated using ImageJ by investigating whole atrial cross-section fields.

After the same preparation, we used rabbit polyclonal antiCD31 (#ab28364, Abcam, Cambridge, MA, USA) at a 1:50 dilution primary antibody to detect capillary density. Positively stained vascular structures were counted and were related to the atrial myocardial area to calculate the capillary density using ImageJ by investigating whole atrial cross-section fields. The antibody against Cx43 (#ab11370, Abcam) at a 1:2000 dilution was used to detect Cx43 intercellular junctions. We selected areas where cardiomyocytes were oriented longitudinally, and Cx43 density was determined.

### 2.8. Statistical Analysis

Results are expressed as mean ± SEM. The assumption of normal distribution of the data was analyzed using the Shapiro–Wilk test. A two-way analysis of variance (ANOVA) with the factors “Sex” (*p*_sex_) and “Exercise” (*p*_ex_) was performed, and *p*-values were calculated for sex and exercise, and their interaction (*p*_int_) was calculated. Post hoc pairwise comparisons were performed using the Tukey method to determine differences between groups (MCo vs. MEx; FCo vs. FEx). A two-tailed *p* < 0.05 value was considered statistically significant.

## 3. Results

### 3.1. Cardiac Morphological Alterations

**Figure 1:**
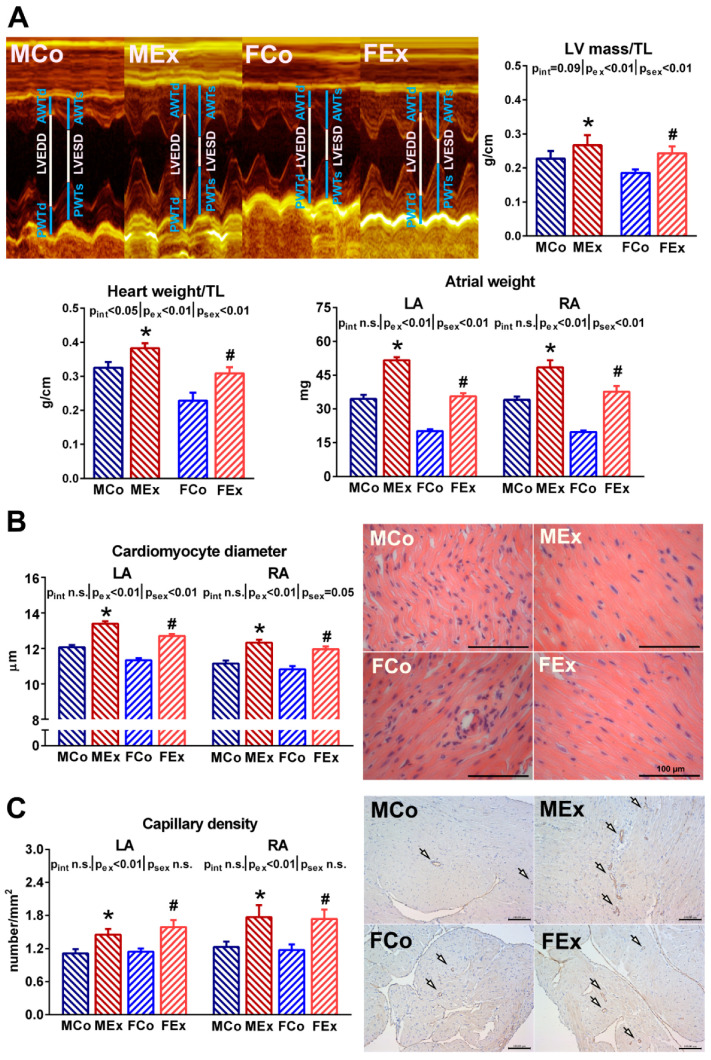
(**A**) Representative M-mode recordings of the left ventricle (LV) from one–one male control (MCo), male exercised (MEx), female control (FCo), and female exercised (FEx) animals. The lines show anterior wall thickness (AWT) at diastole (index: d) and systole (index:s), LVEDD (LV end-diastolic diameter), LVESD (LV end-systolic diameter), and posterior wall thickness (PWT) at diastole (index: d) and systole (index:s). Calculated LV mass and post-mortem measured heart weight values normalized to tibial length (TL) are shown. Atrial weight data from both left (LA) and right atrium (RA) confirmed atrial hypertrophy. (**B**) The upper part shows cardiomyocyte diameter data and representative left atrial hematoxylin-eosin stained sections from one animal of each group (magnification 400×, marker 100 µm). (**C**) The part below shows capillary density data in both atrium and representative CD31 immunostained sections from the left atrium (magnification 200×, marker 100 µm, arrows indicate positively stained capillaries). Values are means ± SEM. * *p* < 0.05 vs. MCo, # *p* < 0.05 vs. FCo. *p*_ex_ and *p*_sex_: *p*-value of two-way analysis of variance (ANOVA) factors exercise and sex, respectively; *p*_int_: interaction *p*-value of ANOVA.

Significant left ventricular hypertrophy in the exercised animals was confirmed by normalized heart weight and LV mass values, respectively (Figure 1A). Female sex was associated with relatively greater ventricular hypertrophy (~25%) than that detected in male counterparts (~15%), compared to respective sedentary controls. Atrial weight data demonstrated significantly increased left and right atrial mass in both male and female animals (Figure 1A). Exercise-induced atrial hypertrophy did not show any sex-specific difference. Our microscopic results were in agreement with the macroscopic ones. The atrial cardiomyocyte diameter was clearly elevated to the same extent in both sexes (Figure 1B). In parallel with the enlargement of cardiomyocytes, we found increased capillary density by CD31 staining in the atrial tissue of exercised animals (Figure 1C).

### 3.2. In Vivo Electrophysiology

**Figure 2:**
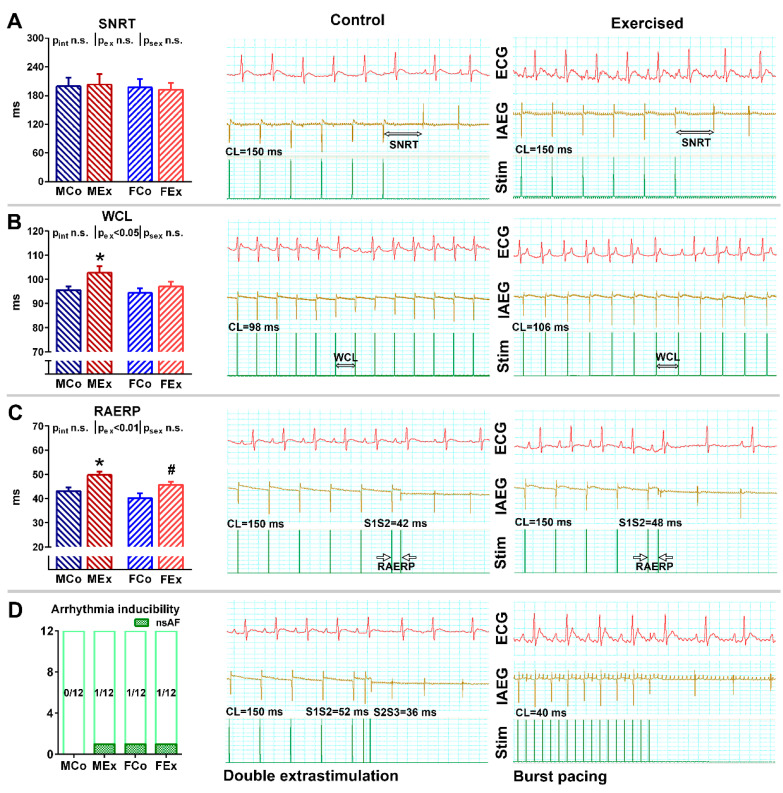
(**A**) Sinus node recovery time (SNRT) data in male control (MCo), male exercised (MEx), female control (FCo), and female exercised (FEx) animals and representative electrocardiogram (ECG, lead II), intraatrial electrogram (IAEG), and stimulation marks (Stim) during the SNRT maneuver in one control and exercised animal. (**B**) Wenckebach cycle length (WCL) data and representative ECG, IAEG, and Stim curves of one control and exercised animal. The longest cycle length (CL) inducing Mobitz type-1 AV-block constituted the WCL. (**C**) Right atrial refractory period (RAERP) data and representative ECG, IAEG, and Stim curves from an exercised and a control animal. (**D**) Atrial arrhythmia inducibility by using double extra stimulation (DES) or burst pacing protocol and representative ECG, IAEG, and Stim curves from an exercised and a control animal. CL: cycle length. Values are means ± SEM. * *p* < 0.05 vs. MCo, # *p* < 0.05 vs. FCo. *p*_ex_ and p_sex_: *p*-value of two-way analysis of variance (ANOVA) factors exercise and sex, respectively; *p*_int_: interaction *p*-value of two-way ANOVA.

According to our results from the resting ECG recordings, exercise training was associated with resting bradycardia (p_ex_ < 0.05 in the overall population), prolonged P wave length and PR interval, as well as with increased amplitude of P wave and QRS amplitude (Table 1). SNRT—after rapid programmed stimulation of the atrium—did not differ between control and trained animals, which suggests an intact function of the sinus node (Figure 2A). WCL was slightly increased as a result of swim training, which was more pronounced in male animals (Figure 2B). We found a significant prolongation of RAERP in exercised male and female rats, which did not show any sex-specific difference (Figure 2C). Arrhythmia inducibility was tested by burst pacing and DES. No sustained atrial arrhythmia was triggered by these programmed stimulations. We could detect very short (<5 s) non-sustained atrial arrhythmias only in the case of three animals (Figure 2D). These data suggest that our training protocol did not result in increased arrhythmia inducibility.

### 3.3. Atrial Oxidative Stress and Tissue Remodelling

**Figure 3:**
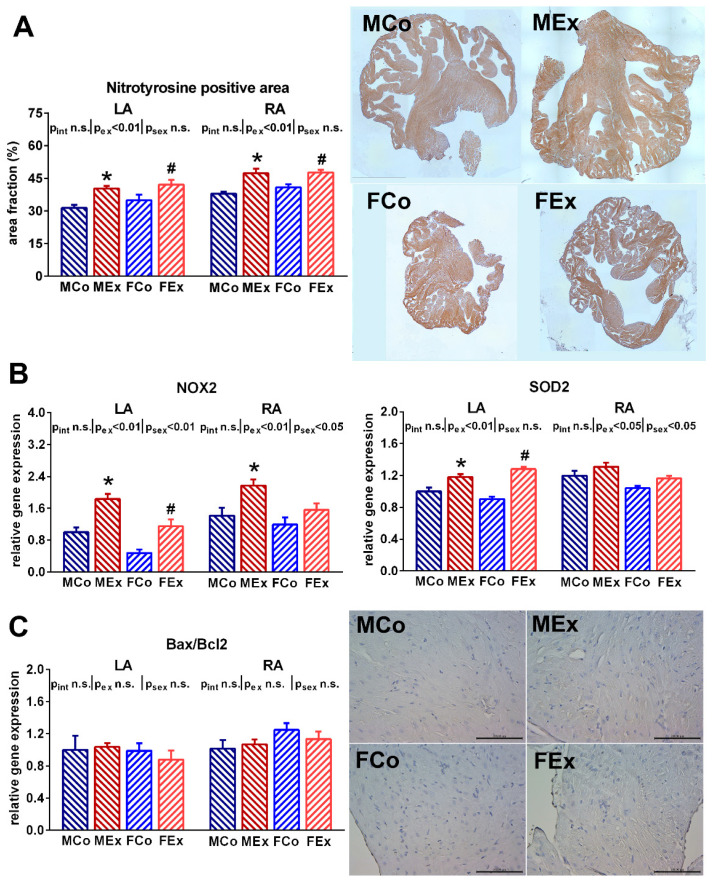
(**A**) Nitrotyrosine positive area fraction data from left (LA) and right atrium (RA) with representative whole left atrial sections from one–one male control (MCo), male exercised (MEx), female control (FCo), and female exercised (FEx) animals. (**B**) Atrial gene expression data of NADPH oxidase 2 (NOX2) and superoxide dismutase 2 (SOD2). (**C**) The atrial gene expression ratio of proapoptotic Bcl-2-associated X protein (Bax) and anti-apoptotic Bcl-2. Representative terminal deoxynucleotidyl transferase-mediated dUTP nick-end labeling (TUNEL) stained left atrial sections (magnification 200x, marker 100 µm). Note that no positively stained nuclei were visualized. Values are means ± SEM. * *p* < 0.05 vs. MCo, # *p* < 0.05 vs. FCo. *p*_ex_ and *p*_sex_: *p*-value of two-way analysis of variance (ANOVA) factors exercise and sex, respectively; *p*_int_: interaction *p*-value of ANOVA.

**Figure 4:**
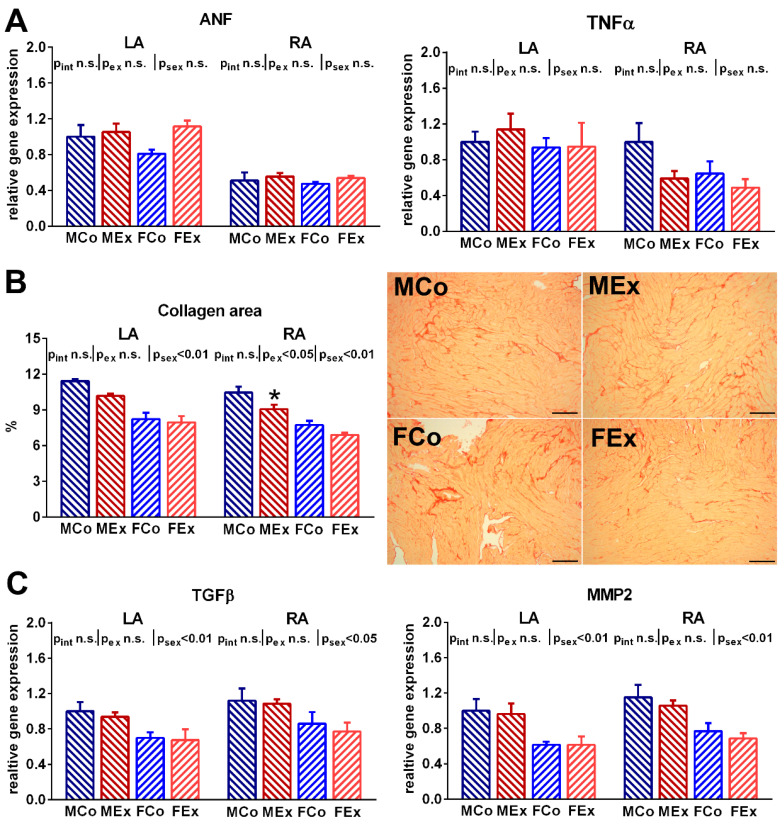
(**A**) Atrial natriuretic factor (ANF) and tumor necrosis factor α (TNFα) gene expression data from left (LA) and right atrium (RA) in male control (MCo), male exercised (MEx), female control (FCo), and female exercised (FEx) animals. (**B**) Atrial collagen area data and representative picrosirius stained LA sections (magnification 200×, marker 100 µm, red stained indicates collagen). (**C**) The atrial gene expression ratio of transforming growth factor β (TGFβ) and matrix metalloproteinase-2 (MMP2). Values are means ± SEM. * *p* < 0.05 vs. MCo. *p*_ex_ and *p*_sex_: *p*-value of two-way analysis of variance (ANOVA) factors exercise and sex, respectively; *p*_int_: interaction *p*-value of two-way ANOVA.

An increased ratio of the nitrotyrosine positive area was detected both in the right and left atrial myocardium of exercised animals, which suggests raised atrial nitro-oxidative stress (Figure 3A). Alongside these alterations, we found increased atrial gene expression of the main contributors of the myocardial antioxidant enzyme system (SOD2, NOX2) in exercised animals (Figure 3B). However, neither TUNEL staining nor the Bax/Bcl_2_ ratio suggested increased apoptotic activity in the atria of exercised rats compared to control ones (Figure 3C). Unaltered ANF and TNFα expression indicated the absence of pathological atrial remodeling and inflammation (Figure 4A). We also examined fibrotic remodeling both in the left and right atrium. Swim training was not associated with increased myocardial fibrotic accumulation or elevated expression of profibrotic factors, such as MMP2 and TGFβ (Figure 4B,C). Female sex was related to a decreased amount of myocardial collagen and decreased gene expression of proteins participating in fibrotic turnover (MMP2 and TGFβ).

### 3.4. Molecular Alterations Related to Electrical Remodeling

**Figure 5:**
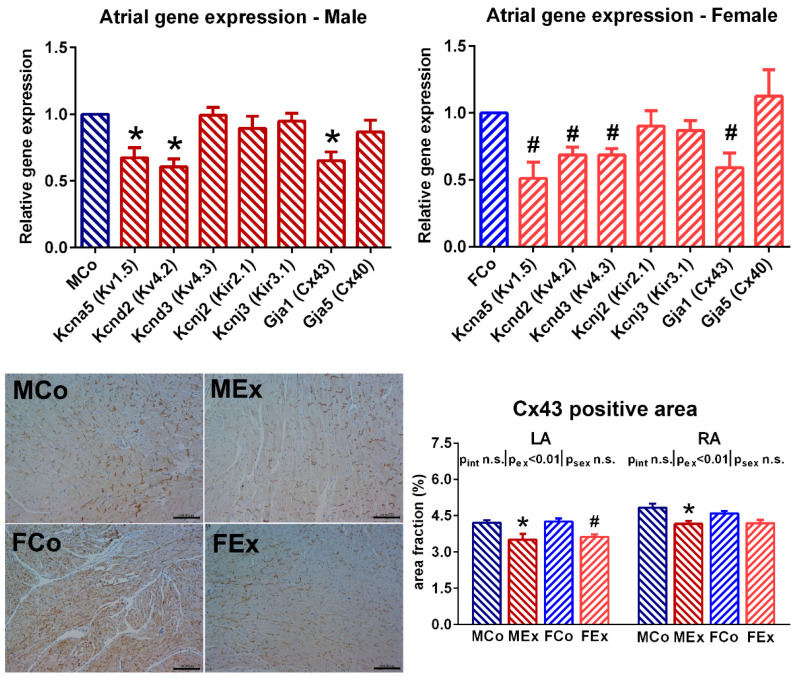
**Upper panel** Right atrial gene expression of different voltage-gated potassium channels (Kv1.5, Kv4.2, and Kv4.3), inward rectifier potassium channels (Kir2.1 and Kir3.1), and connexins (Cx40 and Cx43) in male and female animals. Relative gene expression values of exercised rats were compared to male control (MCo) and female control (FCo) animals. **Lower panel** Representative left atrial Cx43 immunostained sections (magnification 200×, marker 100 µm) from one–one MCo, male exercised (MEx), FCo, and female exercised (FEx) animals a Cx43 density data from the left (LA) and right (RA) atrium. Values are means ± SEM. * *p* < 0.05 vs. MCo, # *p* < 0.05 vs. FCo. *p*_ex_ and *p*_sex_: *p*-value of two-way analysis of variance (ANOVA) factors exercise and sex, respectively; *p*_int_: interaction *p*-value of two-way ANOVA.

We found decreased right atrial gene expression of potassium channels (Kcna5 and Kcnd2 in the case of both genders, Kcnd3 in female animals) in exercised animals compared to controls (Figure 5). The gene expression of Cx43 was also decreased in the exercised rats compared to control ones. Analysis of Cx43 immunostained sections revealed slightly decreased Cx43 density in the atrium of exercised animals (Figure 5).

## 4. Discussion

In this experimental study, a detailed characterization of exercise-induced atrial alterations was provided in a small animal model of an athlete’s heart.

Our previous studies indicated significant physiological left ventricular hypertrophy, which was more pronounced in female rats, along with clear left ventricular functional improvement in rats swim-trained according to our protocol [14,16]. In agreement with these and other observations [17], here we found significant ventricular hypertrophy in both sexes, with a relatively greater enlargement in female animals (Figure 1A). Further, marked atrial hypertrophy at macroscopic and microscopic levels was found that did not show any sex-specific differences. The atrial hypertrophy was comparable to the results of a study, where male animals underwent a 16-week long treadmill training [18]. Although it is difficult to investigate atrial hypertrophy in humans, the dilation of the left atrium was confirmed in highly trained male and female athletes [19,20]. Moreover, our data suggest that exercise training results in a balanced phenotype of hypertrophy, where both left and right atria are adapted to increased volume overload during exercise sessions. The degree of atrial hypertrophy was similar in male and female animals, in contrast to a human study, where less pronounced atrial enlargement was suggested in female athletes compared to male individuals [21]. Experimental studies provide us a more controlled setting to compare the attribution of males and females by eliminating important factors that influence exercise-training response (e.g., differences in training type, quantity, or motivation; control group activity).

Oxidative stress, defined as excess production of reactive oxygen species (ROS), plays a pivotal role in the induction of exercise-induced myocardial hypertrophy, and the Nrf2-mediated antioxidant response can attenuate long-term pathological processes in the myocardium [22]. Moreover, experimental and clinical data indicate that oxidative stress is implicated in the pathophysiology of atrial fibrillation [23]. The present study demonstrated nitro-oxidative stress of left and right atrial tissue in exercised trained rats compared to sedentary controls (Figure 3). The coexisting upregulation of NOX2 and SOD2—as two main antioxidant enzymes in the cardiovascular system—indicates a sustained adaptation of the endogenous antioxidant system in atrial cardiomyocytes to oxidative stress during exercise sessions. This is in line with ventricular data, where exercise-induced oxidative stress has been associated with increased expression of antioxidants [22]. Prolonged oxidative stress is a powerful inducer of cardiomyocyte apoptosis and, therefore, might lead to myocyte loss and fibrotic replacement [24]. TUNEL staining of the atria did not show any DNA fragmentation related to apoptosis. As well, the proapoptotic Bax and anti-apoptotic Bcl-2 gene expression ratio was unchanged in our animals (Figure 3). These data indicate no proapoptotic activity in the atrium of exercised animals. Thus, there is likely a balance between oxidative stress and antioxidant mechanisms. The parallel rise in capillary density also suggests the physiological nature of atrial hypertrophy (Figure 1C).

The volume overload during an aerobic exercise session causes increased stretch of the atrial wall. Sustained volume overload of the heart induces pathological processes, mainly through the formation of ANF, a hormone secreted from the myocardium [25]. ANF gene expression did not differ between control and exercised animals, which were performed in sedentary conditions, at least six hours after the last exercise (Figure 4). This data suggest that atrial mechanical stress during swim exercise did not cause sustained pathological atrial remodeling. Enhanced systemic inflammatory activation was also observed in endurance athletes and experimental animals after prolonged physical activity [26,27]. Additionally, proinflammatory and profibrotic mechanisms play a key role in atrial pathological remodeling and contribute to atrial fibrillation [28]. Both TNFα and TGFβ expression remained unchanged in male and female trained rats, which suggests the absence of these processes in the atria (Figure 4A,C). In line with these data, no collagen accumulation was observed in the atrial myocardium of exercised rats, and MMP2 atrial gene expression also remained unaltered (Figure 4B,C). Other experimental studies in rodents found meaningful collagen accumulation in rats after 16-week long treadmill training and upregulation of fibrotic markers [18,29]. This disparity might be explained by different types, intensity, and length of exercise training, as well as the distinct extent of stress associated with training protocols.

Although training-induced atrial electrical alterations have been characterized by several studies in both athletes and animal models, the findings are not in complete agreement [18,30,31]. This inconsistency could mainly be associated with different exercise protocols and subjects of the investigations. Moreover, an invasive electrophysiology study might not be performed in healthy athletes due to ethical issues. Therefore, most parameters have been obtained from experimental studies. Sinus bradycardia and prolonged WCL might reflect training-induced vagal enhancement, which is a well-recognized consequence of regular training [18,32]. These alterations were also associated with the altered expression of regulators of G protein signaling (RGS) proteins participating in the cardiac regulation of parasympathetic and sympathetic balance [18]. Although vagal type atrial fibrillation has been described and enhanced parasympathetic tone can contribute to AF in athletes, there are also other aspects of electrical remodeling that can influence arrhythmogenicity [33]. In our rat model, prolonged atrial depolarization (PR length) and repolarization (RAERP) were found in both male and female exercised rats compared to control counterparts (Table 1, Figure 4). The exercise-induced alterations of atrial refractoriness have also not been clarified: both increased values on isolated hearts of treadmill-trained rabbits and decreased values during in vivo electrophysiology of rats have been observed [18,31]. It is well-described that AF is maintained by multiple wandering reentrant wavelets activating the atria in a random manner, and longer atrial refractoriness counteracts the proarrhythmic features of the atrial myocardium [34].

The unchanged SNRT and non-inducibility of arrhythmias suggest no pathological atrial electrical remodeling in exercised rats (Figure 4). Our data contradict previous observations, where SNRT was prolonged in treadmill-trained rats and rabbits [18,31]. However, this is in line with human data, where no difference regarding SNRT was observed in athletes undergoing electrophysiological investigation [30]. We could not induce sustained atrial arrhythmia by the application of forceful stimulation protocols (burst pacing and double extra stimulation), which is in line with our other findings: no signs of pathological remodeling in the atria could be detected (Figure 4). Other experimental research groups could induce sustained atrial fibrillation in trained rodents with a similar stimulation protocol [26,31], however, with different types of training.

We found decreased right atrial expression of certain potassium channels participating in the transient outward currents (Kv4.2 and Kv4.3) and in the ultra-rapid activating delayed rectifier current (Kv1.5) (Figure 5). These alterations might provide an explanation of prolonged RAERP; this is in accordance with a study where prolonged action potential duration was observed in swim exercised mice [1]. In addition, these changes might also have an anti-arrhythmic effect as blocking these channels might provide a treatment for atrial fibrillation [35]. The expression of connexins and the density of Cx43 was also investigated because overexpression of Cx43 is one of the main alterations observed in atrial fibrillation [36]. The swim exercise was associated with decreased atrial expression of Cx43, as well as decreased density was observed during histological analysis (Figure 5). This result is also in line with previous findings, where decreased mRNA and protein levels of Cx43 were observed after repeated bouts of swimming exercise [37].

Both male and female rats underwent the same training protocol to detect sex-specific differences in response to exercise training. The electrophysiological alterations were similar in both sexes. According to recently reported data, moderate exercise was protective both in men and women against atrial fibrillation [9,38], while vigorous physical activity was associated with increased atrial fibrillation only in male individuals. Our exercise protocol might provide balanced, long-term training that does not lead to deleterious atrial remodeling in either sex. A characteristic, coherent distinction was observed regarding fibrosis and profibrotic factors: both control and exercised female rats showed significantly decreased collagen area and lower expression of TGFβ and MMP2 (Figure 3). This is in line with previous observations, where women seem to be affected by atrial fibrillation, coronary atherosclerosis, and myocardial fibrosis less than male counterparts [39]. Indeed, there is evidence that estrogen receptors play a role in the regulation of collagen expression of cardiac fibroblasts [40]. Moreover, androgens through their receptors might also regulate cardiomyocyte hypertrophic response [41]. These alterations suggest sex-specific regulation of the connective tissue system in the atrium and form the basis of future studies investigating sex differences in the myocardium.

Our study might serve as an argument for the benign nature of exercise-induced atrial hypertrophy when a balanced training protocol has been accomplished without excessive exercise episodes (such as prolonged endurance races) or performance-enhancing drugs. This is in agreement with the assumed dose-response curve of exercise and the risk of cardiovascular morbidity in athletes [24]. Moreover, the different regulation of atrial fibrotic components might be in the background of the distinct atrial arrhythmia risk in male and female athletes after long-term, extreme physical activity burden [9]. The role of different qualities and quantities, as well as the frequency of repetitive exercise sessions, in the development of exercise-associated atrial fibrillation, should be further investigated.

### Limitations

The interpretation of results from the current study is limited to young rats. The possible influence of age should be assessed in future studies. In addition, the length of the training period and intensity of training sessions might affect the observed phenotype. In vivo investigations (electrophysiology) could be performed under anesthesia, which might have an influence on parameters dependent on the autonomic nervous system, such as heart rate.

Understanding the role of sympathetic and parasympathetic innervation might be difficult under experimental conditions. In these experiments, animals underwent isoflurane anesthesia during electrophysiological studies. Therefore, the values of associated parameters (HR, WCL) might be influenced by this anesthesia. In our previous studies using intravenous anesthetics (pentobarbital and ketamine-xylazine), the heart rate of exercised animals did not differ significantly compared to control ones [32,35]. Proper isoflurane anesthesia provides less influence on heart rate than intravenous anesthetics.

Moreover, rodents and human individuals show a fundamental distinction in the electrical properties of cardiomyocytes, especially the role of calcium ions during repolarization show marked differences.

## 5. Conclusions

In this study, we investigated exercise-induced atrial alterations using a rat model, where a balanced swim training protocol resulted in significant LV physiological hypertrophy and functional improvement. Detailed investigations of atrial function and structure showed physiological myocardial hypertrophy with increased capillary density, lack of pathological processes, such as profibrotic and inflammatory response, and a balance between oxidative stress and antioxidant mechanisms. A decreased expression of potassium channels and Cx43 might contribute to a prolonged atrial effective refractory period. The lack of arrhythmia inducibility suggests benign electrical remodeling.

## Figures and Tables

**Table 1:** Electrocardiogram (ECG) data (lead II). * *p* < 0.05 vs. MCo; # *p* < 0.05 vs. FCo. Data are expressed as mean ± SEM. Two-way ANOVA exercise (*p*_ex_) and sex (*p*_sex_) interaction (*p*_int_).

	MCo (*n* = 12)	MEx (*n* = 12)	FCo (*n* = 12)	FEx (*n* = 12)	*p*_int_	*p*_ex_	*p*_sex_
Heart rate (beat/min)	375 ± 7	355 ± 9	367 ± 5	353 ± 7	n.s.	<0.05	n.s.
P length (ms)	16.3 ± 0.3	17.1 ± 0.3	15.9 ± 0.5	18.4 ± 0.5 ^#^	n.s.	<0.01	n.s.
P amplitude (mV)	0.12 ± 0.01	0.16 ± 0.01 *	0.14 ± 0.01	0.19 ± 0.01 ^#^	n.s.	<0.01	<0.01
PR length (ms)	49.4 ± 0.9	51.9 ± 1.7	49.4 ± 1.1	53.7 ± 1.1	n.s.	<0.05	n.s.
QRS length (ms)	18.2 ± 0.2	18.7 ± 0.3	18.7 ± 0.5	19.0 ± 0.6	n.s.	n.s.	n.s.
QRS amplitude (mV)	0.85 ± 0.05	1.05 ± 0.05 *	1.02 ± 0.05	1.33 ± 0.06 ^#^	n.s.	<0.01	<0.01
QT length (ms)	65.4 ± 3.5	76.7 ± 1.9 *	74.4 ± 2.5	80.9 ± 3.6 ^#^	n.s.	<0.01	<0.05
T amplitude (mV)	0.09 ± 0.01	0.11 ± 0.01	0.11 ± 0.01	0.16 ± 0.02	n.s.	<0.05	<0.05

## Data Availability

The data presented in this study are available on request from the corresponding author.
